# Liquid Crystal Monomers (LCMs) of Emerging Concern: Recent Progress and Challenges in Wastewater Treatment

**DOI:** 10.1007/s40726-025-00377-3

**Published:** 2025-08-11

**Authors:** Sanjeeb Mohapatra, Mui-Choo Jong, Suparna Mukherji, Jules. B. van Lier, Henri Spanjers

**Affiliations:** 1 Department of Water Management, Delft University of Technology, 2628 CN Delft, The Netherlands; 2 Institute of Environment and Ecology, Tsinghua Shenzhen International Graduate School, Tsinghua University, Shenzhen, 518005 China; 3 Environmental Science and Engineering Department, Indian Institute of Technology Bombay, Mumbai, India

**Keywords:** Fluorinated liquid crystal monomers, Wastewater treatment, Sludge, Fate of LCMs, Advanced oxidation processes

## Abstract

**Purpose of Review:**

Liquid crystal monomers (LCMs), used extensively in liquid crystal displays (LCDs), have emerged as persistent, bioaccumulative, and toxic organic pollutants. A network analysis of SCOPUS data revealed significant knowledge gaps, especially concerning the fate of LCMs in WWTPs. The available literature highlights that influent LCM concentrations vary widely, with elevated levels linked to industrial and e-waste recycling activities. This review examines the occurrence, fate, and treatment of LCMs, particularly fluorinated LCMs (F-LCMs), in wastewater treatment plants (WWTPs).

**Recent Findings:**

Conventional WWTP processes achieve moderate removal efficiencies (~ 84%) for LCMs, but F-LCMs often persist. Advanced treatment techniques such as UV/peroxydisulfate (UV/PDS) showed removal rates of 77–84% for LCMs with biphenyl and ethoxy groups. These groups alter electron distribution, making the molecules more susceptible to oxidative attack by reactive species such as hydroxyl and sulfate radicals. Degradation pathways include cleavage of biphenyl, ethoxy, and C-F bonds, producing less toxic by-products such as oxalic acid and cyclohexane. However, some degradation intermediates formed are toxic, necessitating further research of the treatment processes.

**Summary:**

This review underscores the need for systematic monitoring of LCMs in wastewater and their transformation products in treated wastewater and sludge, alongside advancements in treatment technologies to mitigate environmental and health risks. This review highlights the urgency of improving wastewater management strategies for LCMs and the need for future research to address the critical knowledge gaps.

**Supplementary Information:**

The online version contains supplementary material available at 10.1007/s40726-025-00377-3.

## Introduction

Since their discovery in 1888 by an Austrian botanist Friedrich Reinitzer, liquid crystals are increasingly used in material science, biomedical applications, nanoscience, solar cells, smart windows, elastomers, biosensors, and porous membranes [1–3]. At more advanced levels, liquid crystals are also used in head-up displays for automobiles, augmented reality (AR) and virtual reality (VR) systems, and fifth- and sixth-generation (5G/6G) telecommunications [4–6], and even in quantum computing [7]. The global liquid crystal display (LCD) market, valued at USD 152.6 billion in 2021, is projected to reach approximately USD 3.33 trillion by 2032, growing at a compound annual growth rate (CAGR) of 32% [8]. However, the widespread use of liquid crystal-based products raises concern about electronic waste containing liquid crystals, as the amount of such waste is expected to multiply significantly in the environment. A 2024 report by the United Nations Institute for Training and Research (UNITAR) revealed that global e-waste production has surged by over 82% between 2018 and 2022, reaching 62 million tons in 2022 [9]. Within this, 214 tons of liquid crystal monomers (LCMs), the building blocks of LCDs, is discarded annually, potentially contaminating the environment.

Liquid crystals typically have a rigid core composed of two benzene rings, connected directly or through an intermediate group, which gives the molecule its basic anisotropic structure [10]. This structure, known as the “monomeric unit” or “monomeric mesogen,” or “LCM,” forms the fundamental building block of liquid crystals. Compared to polychlorinated or polybrominated biphenyls (PCBs/PBBs), which are also traditionally associated with electronic devices, the structure of LCMs is more complex and is generally characterized by cyano (CN-LCMs), fluorine (F-LCMs), chlorine (Cl-LCMs), and bromine (Br-LCMs) functional groups on the phenyl ring, although some LCMs may lack these functional groups entirely (non-LCMs). The increased use and subsequent disposal of liquid crystals may pose significant environmental and human health risks [11–14], potentially comparable to or greater than those presented by structurally analogous PCBs/PBBs, polycyclic aromatic hydrocarbons (PAHs), polybrominated diphenyl ethers (PBDEs), polyfluoroalkyl substances (PFAS), and other contaminants [15–17].

**Fig. 1:**
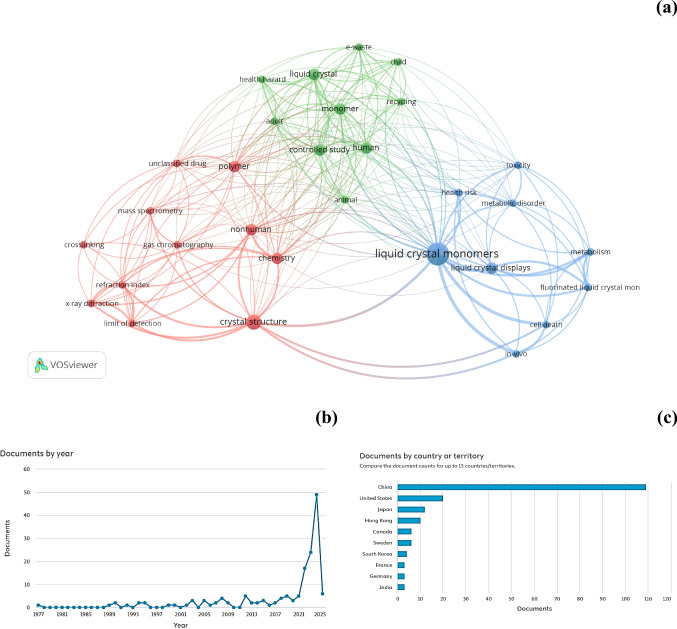
Based on the SCOPUS database, (**a**) a network analysis of literature on liquid crystal monomers (LCMs), (**b**) the annual number of articles published up to 2025, and (**c**) the countries with the highest publication counts

A search of the SCOPUS database using the keywords “liquid,” “crystal,” and “monomers,” restricted to the “environmental sector,” yielded only 155 articles, of which 139 were research articles. A network analysis (Fig. 1a) of these data revealed three distinct clusters, with dominant keywords “liquid crystal monomers” and “monomers.” Among these, “fluorinated liquid crystals” frequently appeared alongside “gas chromatography,” a common analytical method for quantifying LCMs [18, 19].

Another significant cluster highlighted the toxicity of LCMs, with studies conducted in laboratory-scale controlled environments and investigations into exposure risks for vulnerable populations, such as children working or living in e-waste recycling sites [11–14]. Using the Toxicity Estimation Software Tool (TEST), several F-LCMs were found to exhibit high acute and developmental toxicity in rats, with oral LD_50_ values ranging from 41.86 to 562.43 mg/kg and developmental EC_50_ values between 0.70 and 1.08 mg/kg [20]. Furthermore, in silico evaluations highlighted high developmental toxicity (0.41–0.85 mg/L) and mutagenicity (0.06–0.53 mg/L) for LCMs and their degradation products, underscoring concerns beyond acute effects [21]. Long-term exposure studies using zebrafish models have revealed severe morphological, behavioral, and hormonal disruptions. Exposure to six common F-LCMs led to deformities, inhibited phototactic behavior, and disrupted thyroid hormone synthesis, signalling pathways, and vision-related gene expression [14]. These effects were observed at environmentally relevant concentrations ranging from 0.1 to 300 ng/L, highlighting the endocrine-disrupting potential of LCMs at trace levels. In a separate study, ecotoxicity profiling of 12 F-LCMs revealed that most were “very toxic” to aquatic species, with LC_50_ values < 1 mg/L for fathead minnow and daphnia (e.g., LC_50_: 0.026–7.1 mg/L for fish; 0.15–0.89 mg/L for daphnia) [21]. Again, the degradation products remained detrimental, falling into the “toxic” category (1–10 mg/L). Similar classifications were made by ECOSAR-based assessments for 4-cyano-3,5-difluorophenyl 4-ethylbenzoate (CEB-2F) and its transformation products, which showed LC_50_/EC_50_ values between 1.29 and 4.18 mg/L for green algae, daphnia, and fish [22]. Chronic toxicity was also noted, with chronic values (ChV) below 1 mg/L for green algae and fish and 1.60 mg/L for daphnia. Notably, F-LCMs were found to be associated with health risks, including cell death (based on cell line studies).

Further analysis of the 155 documents showed that the highest number of articles (*n* = 49) was published in 2024 (Fig. 1b). China led with the highest number of publications (*n* = 109), followed by the USA (*n* = 20), Japan (*n* = 12), and Hong Kong (*n* = 10) (Fig. 1c) for the entire search period. In China, one of the major producers of LCDs, the total quantity of liquid crystal inventory is expected to reach nearly 11,000 tonnes by 2025 [23]. E-waste dismantling in China alone releases between 1 and 107 kg of LCMs per year [24] from the discarded computer monitor and television LCD panels through run-off, air emissions, and dust [25, 26]. A few studies have reported LCM contamination in sediments [27–29], soil [30], and landfill leachate [31] in China.

While LCMs are increasingly recognized as emerging pollutants due to their persistence, bioaccumulative potential, and environmental toxicity [27–29], the network analysis did not capture keywords such as “water” and “wastewater,” highlighting a significant knowledge gap in this domain. To address this, further article search was conducted using additional keywords “wastewater,” “water,” and “treatment” to identify articles reporting the occurrence and fate of LCMs in wastewater treatment plants (WWTPs) and water treatment plants (WTPs). Only two studies have reported the occurrence of LCMs at full-scale WWTPs; five studies have reported UV treatment for LCM removal, whereas there were no studies on LCMs in WTPs. LCMs enter WWTPs through urban domestic wastewater, industrial effluents from LCM production facilities, and e-waste recycling facilities. Despite their ubiquity, the occurrence, fate, and treatment of LCMs remain poorly understood. The reviews conducted so far have primarily focused on persistence [32, 33], aquatic toxicity (Table S1), and human health risks associated with LCMs (Table S1) [13, 33–36]. Despite these concerns, relatively little attention has been paid to the removal of LCMs. Once released from WWTPs, these contaminants can enter the environment and potentially re-enter human systems through drinking water sourced from polluted environments. Therefore, it is essential to understand their fate in both WWTPs and WTPs in order to implement appropriate advanced treatment techniques and reduce potential human and ecological health risks. Thus, this review aims to provide an overview of the occurrence and fate of LCMs at WWTPs based on the available literature covering around 65 LCMs, their fate during different treatment stages, and the effectiveness of treatment techniques such as UV radiation for their degradation. Where literature data on LCMs were limited at different stages of treatment, the fate of LCMs was explained using structurally similar compounds. Identifying knowledge gaps and proposing recommendations for future research on LCM removal during wastewater treatment was one of the key objectives of this review.

## Occurrence at WWTPs

### Occurrence in the Aqueous Phase

**Fig. 2:**
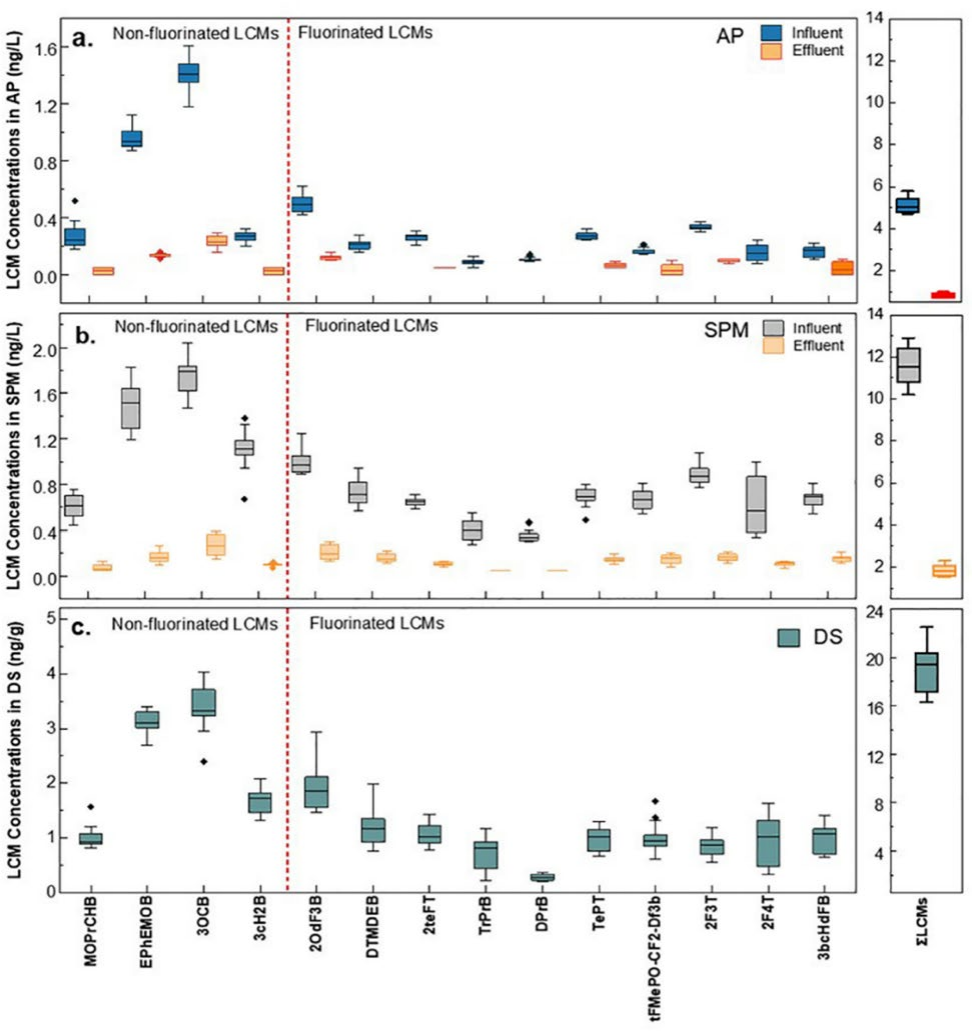
Concentrations of LCMs in (**a**) the aqueous phase (AP), (**b**) suspended particulate matter (SPM) from influent and effluent samples (ng/L), and (**c**) dewatered sludge (DS) samples (ng/g) (reused with permission from Zhan et al. [38])

Two studies have so far reported the occurrence and fate of LCMs in WWTPs in China, providing key insights into their occurrence and fate [37, 38]. LCMs enter municipal WWTPs through diverse pathways, including urban domestic wastewater, industrial effluents, and e-waste recycling facilities. Influent total concentrations of LCMs varied between 5 and 17 ng/L, depending on demographic and industrial factors (Fig. 2) [38]. Among the LCMs studied, 10 fluorinated LCMs (F-LCMs) were present at a total concentration of 8.90 ± 0.10 ng/L. Non-fluorinated LCMs (NF-LCMs) such as 4′-propoxy-4-biphenylcarbonitrile (3OCB) and F-LCMs like 1-ethoxy-2,3-difluoro-4-(4-propylphenyl)benzene (2OdF3B) were the most prevalent. The composition of influent LCMs demonstrates the diversity of these compounds. Urban centers with high electronic device usage and e-waste generation exhibit higher influent LCM levels [38]. In regions such as East China, the influent concentration of LCMs was elevated due to proximity to large industries.

The treatment train at the WWTP included a screen chamber, primary sedimentation tank, aeration tank, secondary sedimentation tank, UV disinfection, and sludge dewatering process. For the full form of the abbreviations, refer to Table S2.

The effluent concentration of LCMs ranged between 1 and 3 ng/L, representing a removal efficiency of over 80% compared to the influent levels [38]. Among the LCMs, F-LCMs were least removed, with the highest removal (7%) observed for 2OdF3B within the F-LCMs. The total annual emission of LCMs from a single WWTP was estimated at 3.04 kg, with most compounds adsorbed onto particulate matter (69%) [38]. These particulates can accumulate in aquatic ecosystems, leading to bioaccumulation in aquatic organisms. In addition, dissolved LCMs (31%) are more likely to disperse in water bodies, contributing to widespread contamination. This discrepancy necessitates further research into the distribution of LCMs in the solid phases such as sludge at WWTPs. The estimated annual emission of total LCMs (3 kg/year) [38] indicates that the previous global emission estimate from TV and computer LCD panels (1–107 kg/year) may have been underestimated [24].

### Occurrence in Sludge

**Fig. 3:**
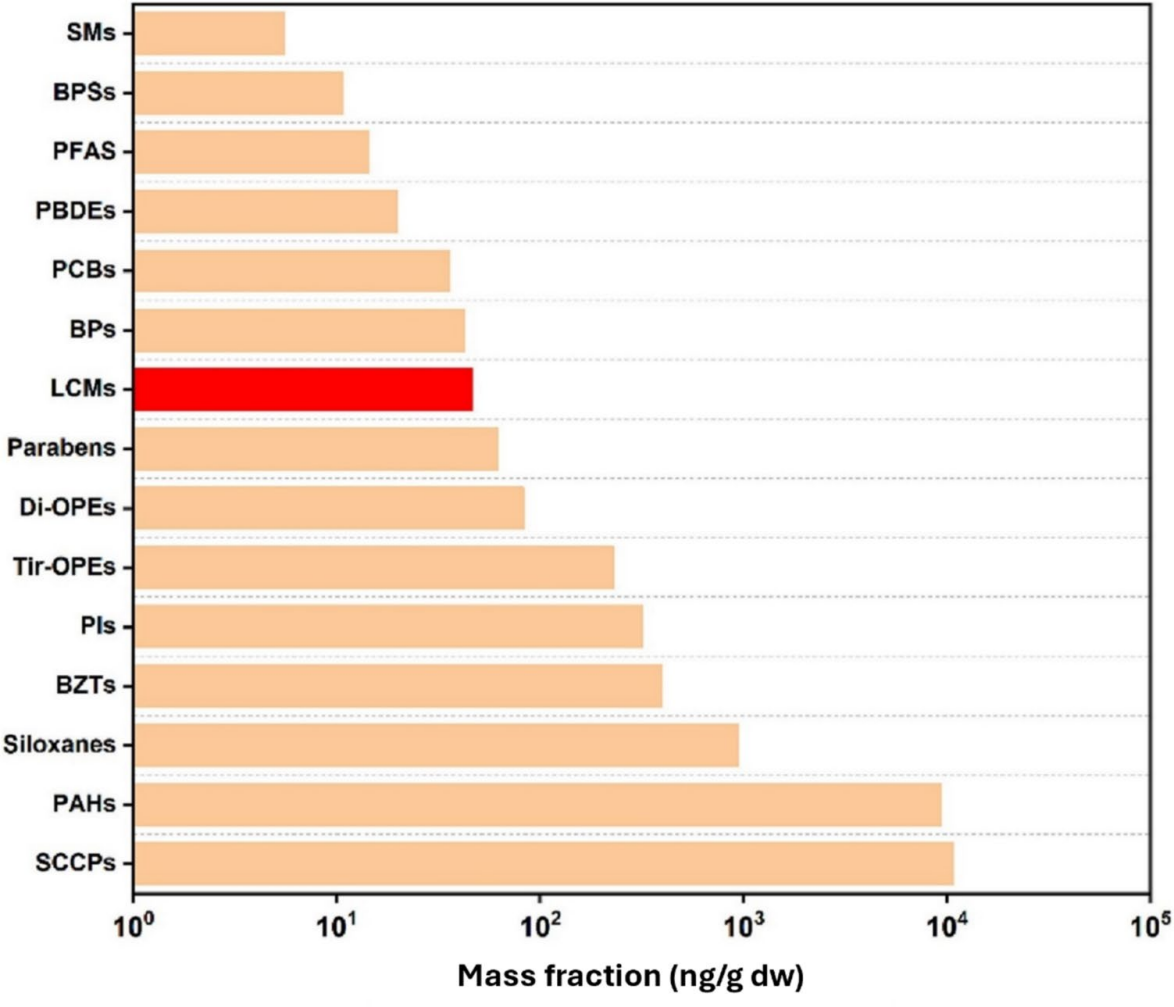
Distribution of LCMs and other contaminants in sewage sludge in China (reproduced with permission from Feng et al. [37]). Abbreviations: SMs (synthetic musks), BPSs (bisphenol S analogs), PFASs (perfluoroalkyl substances), PBDEs (polybrominated diphenyl ethers), PCBs (polychlorinated biphenyls), BPs (bisphenol analogs), Di-OPEs (organophosphate diesters), Tri-OPEs (organophosphate triesters), PIs (photoinitiators), BZTs (benzotriazole UV stabilizers), PAHs (polycyclic aromatic hydrocarbons), and SCCPs (short-chain chlorinated paraffins). The mass fraction of contaminants is expressed in logarithmic units

Sewage sludge acts as a major sink for hydrophobic LCMs, with the total mass fraction of LCMs ranging from 17.2 to 225 ng/g [37, 38]. The concentrations of the LCMs studied were slightly higher than those of structurally similar PBDEs and PCBs in China (Fig. 3). The hydrophobic LCMs, particularly those with a biphenyl (BA) backbone, dominate in sludge due to their strong adsorption affinity. BAs account for 78% of the total LCMs in sludge, with fluorinated biphenyls (FBAs) contributing to the remaining 22% [37]. Regional variations in sludge LCM concentrations are notable. East China exhibits higher levels (median LCM level 59 ng/g) due to proximity to industrial and e-waste recycling facilities, whereas West China showed lower levels (median LCM level 29 ng/g). Factors influencing these regional differences include the density of industries, the prevalence of LCD manufacturing, and the e-waste management practices. In a separate study, 3OCB and 1-(2-(4-ethylphenyl)ethynyl)−4-methoxybenzene (EPhEMOB) were found to be the predominant NF-LCMs detected in the wastewater suspended particulate matter samples, whereas 2OdF3B was identified as the most abundant F-LCM [38]. These results corroborate with the distribution of 3OCB, EPhEMOB, and 2OdF3B observed in the dewatered sludge samples. Although a decrease in LCMs was observed across different stages of treatment in the solid phases, their presence in sludge poses significant long-term environmental risks. Thus, the disposal of sludge through land application may have detrimental consequences.

## Fate and Treatment in WWTPs

### Fate in the Aqueous Phase During Different Stages of Wastewater Treatment

**Fig. 4:**
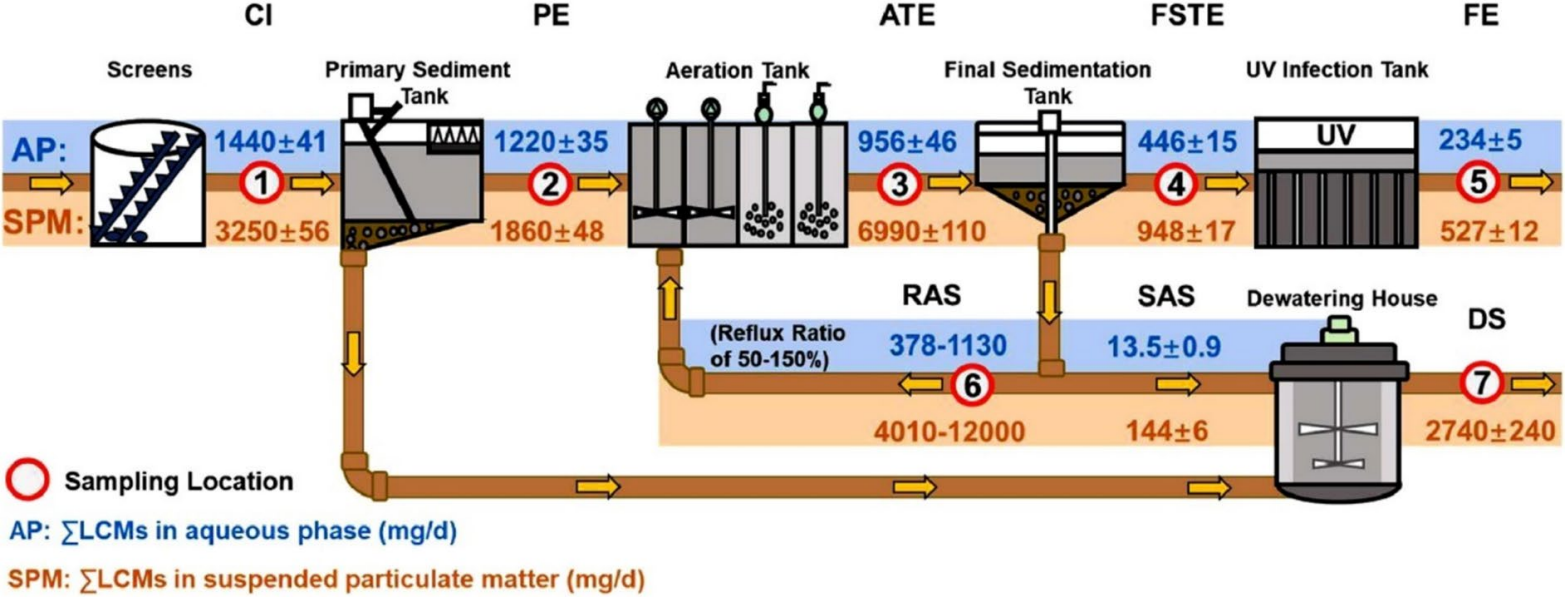
Mass flow (mg/day) of LCMs during different stages of wastewater treatment (reused with permission from Zhan et al. [38]). Abbreviations: CI, raw influent; PE, primary effluent; ATE, aeration tank effluent; FSTE, final sediment tank effluent; FE, Final effluent; RAS, return activated sludge; SAS, surplus activated sludge; DS, dewatered sludge)

LCMs undergo a multi-stage treatment process in WWTPs with an overall removal efficiency of 84% from the aqueous phase (Fig. 4) [38]. A typical full-scale conventional WWTP operates through multiple stages: preliminary, primary, secondary, and tertiary treatment [39, 40]. The preliminary and primary stages involve processes, such as screening, grit removal, and primary clarification, during which chemical coagulation or lamella settling can influence the removal efficiency of LCMs. WWTPs equipped with lamella settlers could achieve higher removal efficiencies for both LCMs and total suspended solids (TSSs) due to enhanced surface loading rates. However, operational issues such as short-circuiting, temperature differences, and short retention time at high flow rates can compromise clarification, leading to elevated LCM concentration in the primary clarifier effluent. Optimizing hydraulic retention time (HRT) and solid retention time (SRT) in a sedimentation tank promotes TSS removal, which can also significantly enhance LCM removal due to the hydrophobic nature of LCMs. Biphenyls and their derivatives, and common LCMs, also exhibit a high sorption potential. Thus, 34% of the LCMs were removed during primary sedimentation through adsorption onto suspended solids [38].

After primary treatment, the LCM-contaminated wastewater undergoes secondary treatment, where biological treatment is achieved in the activated sludge process or membrane bioreactors. In this step, LCMs are often poorly removed due to their extremely low concentrations as well as stable structure, persistence, bioaccumulation tendencies, and toxicity. For instance, a study by Zhan et al. reported that only 16% of LCMs were removed through biodegradation [38]. In another study, secondary treatment processes contributed to only 25% LCM removal [37]. High-molecular-weight LCMs, particularly fluorinated ones, exhibit lower removal efficiency due to low solubility, poor bioavailability, and strong adsorption to particulates, as observed for PAHs [41–43]. Some of these F-LCMs include 3,4,5-trifluoro-4′-(trans-4-propylcyclohexyl)biphenyl (TrPrB) and 3,4-difluoro-4′-(trans-4-propylcyclohexyl)biphenyl (DPrB), whereas aerobic biodegradation effectively degraded NF-LCMs characterized by functional groups such as alkoxy or alkyl groups (e.g., 3OCB), owing to their lower chemical stability [38]. However, co-metabolism of LCMs bound to organic matter may occur during biological treatment, although co-metabolism of LCMs has not been explored adequately. Low mixed liquor-suspended solid (MLSS) concentrations of 2000 mg/L have significantly enhanced the removal of structurally similar contaminants such as PBDEs, regardless of HRT [44]. Conventional activated sludge systems operating with MLSS concentrations above 2000 mg/L, HRTs of at least 10 h, and SRTs of at least 9 days are expected to achieve optimal removal of LCMs [44]. Conversely, suboptimal operational efficiency may lead to higher LCM concentration in secondary effluent streams.

Advanced treatment processes for biological nutrient removal (BNR) use various bioreactors, such as anaerobic–anoxic–aerobic or anoxic–anaerobic–anoxic–aerobic bioreactors, which are operated under prolonged HRT and SRT. Therefore, BNR processes could potentially enhance LCM removal under optimized conditions, as has been observed for structurally similar compounds. However, such processes are often not practiced in underdeveloped and developing regions, where the use of aerated or facultative lagoon-based systems is predominant. Lagoon-based systems, characterized by high HRTs (ranging from 20 days to 6 months), can achieve significant LCM removal due to the extended retention time, although the performance is sensitive to diurnal and seasonal temperature variations. For example, microbial activity and photodegradation are more effective in summer, and this could lead to higher removal of aqueous phase LCMs compared to winter. Similar seasonal trends have been observed for PBDEs, with removal efficiency declining from summer to winter [44]. However, even with UV treatment, the overall LCM removal efficiency was limited. For instance, a full-scale WWTP reported only 84% mean removal for 64 LCMs [38]. UV treatment alone contributed to 7% only, highlighting the stability and persistence of LCMs. At the same time, the removal of LCMs during UV treatment is also negatively impacted by the presence of particulate matter. The study further confirmed that a significant fraction of LCMs, particularly F-LCMs, remain in the treated effluent [38]. These compounds are distributed between the liquid and the solid phases, with approximately 69% adsorbed onto solids, necessitating the urgency to address their fate in the sludge. Notably, biodegradation simulations revealed that transformation products of F-LCMs, such as defluorinated biphenyls, can persist in the environment at levels equal to or even exceeding those of the parent compounds [37].

### Fate in the Sludge During Different Stages of the Treatment

Similar to the variations in LCM concentration, as well as their fate and treatment in wastewater, analyzing and comparing the distribution of LCMs across different sludge samples during different treatment stages are crucial, as sludge serves as a sink for a large fraction of LCMs. This can be explained by determining their distribution coefficients (*K*_d_). This coefficient, defined as the ratio of LCM fraction in solids (ng/kg) to their fraction in the liquid phase, is expected to vary widely due to differences in the characteristics of primary sludge, waste biological sludge, and dewatered sludge generated from the primary clarifier, secondary clarifier, and sludge treatment units, respectively [45]. Chemically assisted primary clarifiers will possibly show more efficient LCM separation from wastewater compared to conventional clarifiers, as has been observed for structurally similar PBDE compounds. This hypothesis is based on the higher log *K*_d_ values for LCMs in primary clarified sludge. Conversely, lower halogenated congeners of LCMs, with low *K*_d_ values, are more likely to be dispersed into the aqueous phase and colloidal solids that escape through the primary effluent. Factors such as molecular interactions, solubility of LCMs, and the properties of associated solid substances play a significant role in their distribution in the solid and aqueous phases. For example, LCMs with low halogenation and high octanol–water partition coefficient (*K*_ow_) values might show greater affinity for the aqueous phase. In contrast, high molecular weight LCM congeners might bind more readily to particulate matter despite their low *K*_ow_ values. Similar findings have been reported for chlorinated benzenes, despite their relatively low log *K*_ow_ values [46]. Given the limited experimental data on log *K*_d_ values for LCMs across different sludge types [19], it is crucial to determine these values and establish correlations with log *K*_ow_ and the molecular weight distribution. Optimizing operational parameters, such as extending HRT, may enhance solid–liquid separation and increase LCM partitioning into solids.

As wastewater progresses from the primary to the secondary clarifier, LCMs are expected to partition more into suspended solids [38]. Possibly, this shift occurs because LCMs initially bind to dissolved organic matter in the influent wastewater but subsequently redistribute to suspended solids as the concentration of dissolved organic matter decreases during treatment [47]. The hydrophobic nature of biomass in secondary sludge further promotes the partitioning of LCMs to secondary sludge. Consequently, LCMs that escape primary clarification will likely be captured by biomass and accumulate in secondary sludge, as observed for other contaminants such as alkylphenolic surfactants and PBDEs [48]. Additionally, high MLSS concentrations and extended SRTs have been shown to enhance LCM partitioning to secondary sludge, similar to trends reported for PBDEs and nonylphenol polyethoxylates [45, 48].

The regular application of treated sludge as fertilizer may lead to LCM accumulation in crops [49], underscoring the need to monitor these compounds in various sludge types and sludge treatment processes.

## Fate of LCMs During UV and Advanced Oxidation Treatment

UV treatment is employed as a tertiary wastewater treatment step and primarily serves as a polishing step in WWTPs. Five studies have so far reported the photodegradation of LCMs under varying experimental conditions, providing key insights into UV-mediated degradation efficiencies and mechanisms. The degradation of five cyano LCMs was investigated under high-energy UV light at a wavelength of 254 nm [50]. However, these experiments were conducted at relatively high concentrations of LCM (5 mg/L), which limits their environmental relevance (Table 1). Despite this, the study incorporated humic acid (HA) as a model for dissolved organic matter (DOM) to explore the role of direct and indirect photodegradation. Under UV irradiation, non-cyano LCMs can be excited to singlet or triplet states, facilitating electron transfer and reactive oxygen species (ROS) generation. Singlet oxygen (^1^O_2_) and superoxide radicals (O_2_^•−^) were found to enhance degradation, particularly for compounds like 4-(trans-4-vinylcyclohexyl)benzonitrile (2eCHB), where double bonds promoted exciton separation and electrophilic attack by ROS. In contrast, cyano LCMs, such as 2OCB, with ethoxy groups, were less susceptible to direct photodegradation. Theoretical calculations further indicated that the benzene ring, ethoxy groups, and double bonds significantly influence the electron distribution in cyano LCMs. Wu et al. [51] extended this photodegradation research to a fluorinated LCM (4-cyano-3-fluorophenyl 4-ethylbenzoate, CEB-F). The experiments were conducted at low initial concentrations of CEB-F under UV lamps with a wavelength range of 200–1000 nm, simulating natural environmental conditions. Quenching studies confirmed the critical role of superoxide radicals (O_2_^•−^) in breaking the strong fluorinated bonds, while hydroxyl radicals (^•^OH) played a minor role. CEB-F degraded into defluorinated intermediates through ROS-mediated pathways, including hydroxylation and C-O/C-F bond cleavage [51]. These findings highlight the importance of optimizing UV photolysis conditions, such as exposure duration and light intensity in treatment plants, for the effective degradation of F-LCMs. For the first time, Yang et al. investigated the degradation of 23 LCMs, belonging to the biphenylethyne, phenylbenzoate, and diphenyl/terphenyl classes [52]. The experiments were conducted at a low initial concentration of each LCM (2 µM) to mimic prevalent environmental levels, using a 500-W high-pressure mercury lamp with 290-nm cutoff filters. Quenching experiments revealed that ^•^OH and singlet oxygen (^1^O_2_) play key roles in driving degradation, with self-sensitized photolysis being particularly significant for diphenyl/terphenyl LCMs. In their experiments, biphenylethyne LCMs underwent degradation primarily through oxidation and cleavage of the alkynyl groups, making biphenylethyne LCMs more susceptible to photodegradation compared to the other classes. Diphenyl/terphenyl LCMs exhibited significant degradation via self-sensitized photolysis mechanisms, including the oxidation of the benzene rings and cleavage of the molecular chains. In contrast, phenylcyclohexane LCMs demonstrated notable resistance to photodegradation. This resistance can be attributed to the reduced availability of electron-donating sites in their molecular structure, thereby restricting interactions with reactive oxygen species. When the study was extended to investigate the impact of Suwannee River fulvic acid (SRFA, 9 mg/L), commonly used as a model for DOM, on the degradation of the phenylbenzoate LCMs, strong inhibitory effects were observed [53]. These effects were attributed to the competitive absorption of light by fulvic acid and the quenching of the excited states of the LCMs [53–55]. Thus, it can be said that if tertiary-treated wastewater effluent contains organic matter or suspended solids, UV treatment will not be very effective in removing LCM. Thus, only 7% removal was achieved during full-scale WWTP UV-based treatment [38]. In contrast, nitrate (NO_3_^−^) and bicarbonate (HCO_3_^−^) promoted photodegradation of phenylbenzoate LCMs by generating additional ROS. Moreover, the photolysis products exhibited greater toxicity than the parent LCMs, particularly products with increased -OH or fluorine atoms, as confirmed by toxicity assessments using *Vibrio fischeri* and molecular docking studies [49].

**Table 1:** An overview of experimental conditions such as target LCMs, their concentration, light sources, experimental duration, and quenching studies for photochemical oxidation studies

Parameter	Cyano LCMs	Fluorinated LCMs	General LCMs	Phenylbenzoate LCMs
Target LCM compounds	2CB, 4CB, 2OCB, 2CHB, 2eCHB	4-Cyano-3-fluorophenyl 4-ethylbenzoate	23 LCMs (4 structural classes)	CE, CE-F, CE-2F
Experiment matrix	Acetonitrile:water (80:20)	Phosphate buffer (10 mM, pH 7.4)	Methanol, n-hexane	Water with ≤ 0.5% acetonitrile
Light source	UV lamps (254 nm)	Mercury lamp (300 W, 200–400 nm) Xenon lamp (800 W, 200–1000 nm)	Mercury lamp (500 W, > 290-nm filter)	Mercury lamp (500 W, > 290-nm filter)
Light intensity	75 mW/cm^2^	5 mW/cm^2^	Not specified	Not specified
Reaction volume	20 mL	50 mL	25 mL	100 mL
Reaction duration	80 min	150 min	4 h	80 min
Quenching and other experiments	Effect of humic acid and ROS quenching experiments	ROS quenching experiments	Quenching of triplet excited LCMs, ^•^OH, ^⋅^O_2_^⋅−^, and ^1^O_2_	• Effects of DOM (9 mg/L) and typical anions (Cl^−^, NO_3_^−^, SO_4_^2−^, and HCO_3_^−^) (0.5 mM) and cations (K^+^, Ca^2+^, Na^+^, and Mg^2+^) (0.05 mM)
				• Quenching of triplet excited LCMs, ^•^OH, ^⋅^O_2_^−^, and ^1^O_2_
Reference	[50]	[51]	[52]	[53]

In a separate study, the use of UV coupled peroxydisulfate (PDS) for the degradation of 12 F-LCMs resulted in increased degradation efficiency, especially for 4-ethoxy-2,3-difluoro-4′-(trans-4-propylcyclohexyl)biphenyl (EDPB) with biphenyl and ethoxy groups, and 1-ethoxy-2,3-difluoro-4-[(trans,trans)−4′-propyl[1,1′-bicyclohexyl]−4-yl]benzene (EDPBB), 1-ethoxy-2,3-difluoro-4-(trans-4-propylcyclohexyl)benzene (EDPrB), and 1-[4-(4-butylcyclohexyl)cyclohexyl]−4-ethoxy-2,3-difluoro-benzene (BCEDB) with ethoxy groups, achieving removal efficiencies ranging from 77 to 84% [21]. LCMs with biphenyl and ethoxy groups exhibit the highest degradation, as these groups significantly alter electron distribution, making the molecules more susceptible to oxidative attack by ^•^OH and SO_4_^•−^ radicals. The defluorination efficiency was higher for LCMs with fewer and less symmetrical C-F bonds. The degradation pathways involved the cleavage of biphenyl, ethoxy, and C-F bonds, producing oxalic acid and cyclohexane as primary by-products. Importantly, the UV/PDS treatment reduced the acute and developmental toxicities of the F-LCMs, as the degradation products were less harmful than the parent compounds. However, it is worth mentioning that the efficiency of the UV/PDS system can be significantly reduced in real wastewater matrices. For example, Miklos et al. investigated UV/PDS performance in treated municipal wastewater, revealing that the degradation efficiency of trace organic chemicals is significantly influenced by the composition of dissolved organic matter (DOM) and inorganic constituents [56]. Sulfate radicals (SO_4_•−), generated via UV activation of PDS, were shown to be selective oxidants that compete with DOM for reactivity. Lower dissolved organic carbon (DOC) content resulted in improved radical availability and contaminant removal. Complementing these findings, Tang et al. confirmed that matrix constituents such as bicarbonate, carbonate, chloride, and nitrate significantly suppress radical activity by acting as scavengers or competing for SO_4_^•⁻^ [57]. Higher initial humic acid concentration commonly encountered in WWTPs can reduce the removal of LCMs due to limited radical availability and increased light absorption/scattering by organic matter [54, 55, 58].

So far, only one study focused on the degradation of 4-[difluoro(3,4,5-trifluorophenoxy)methyl]−3,5-difluoro-4′-propylbiphenyl (DTFPB) using synchronized oxidation-adsorption (SOA) Fenton technology, achieving 94% removal of DTFPB along with partial mineralization [59]. Optimized conditions included a pH range of 2.5–3.0, an Fe^2+^/H_2_O_2_ molar ratio of 1:4, and the coupling of ^•^OH oxidation with adsorption on ferric hydroxide particles. Three degradation pathways involving eight intermediate products were proposed. The SOA process was not significantly affected by various ions other than carbonate and phosphate ions, which reduced the oxidation efficiency.

## Conclusions

LCMs have been detected in two municipal WWTPs, with influent concentrations linked to urbanization and industrial activities. Their presence in wastewater and sludge underscores the challenges posed by these persistent pollutants in wastewater management. While conventional treatment achieves moderate removal efficiencies for non-LCMs, F-LCMs tend to persist due to their hydrophobicity, chemical stability, and resistance to biodegradation. The partitioning of LCMs between sludge and wastewater emphasizes the need for improved treatment technologies and sludge management practices. The introduction of advanced treatment methods, such as UV/PDS, has demonstrated promise, particularly for compounds like EDPB and EDPBB, which exhibit enhanced degradation and detoxification in lab experiments. However, AOPs require careful consideration due to their potential high energy and operational costs. Scaling up AOPs, particularly in under developed countries that struggle to meet basic nutrient removal standards, faces significant challenges, including the high costs of chemicals and energy, the complexity of integrating them into existing infrastructure, frequent maintenance, and the need for advanced monitoring and control systems.

To effectively combat the environmental and health risks posed by LCM pollution, future efforts must prioritize:

- Comprehensive monitoring studies on the occurrence and fate of LCMs as well as their transformation products at WWTPs employing different treatment technologies [40, 60–62] at a global scale.
- The creation of a database of novel LCMs and their transformation products, along with a standardized identification protocol for accurate quantification of these compounds in wastewater, sludge, and water matrices.
- As LCMs resist biodegradation and UV-based degradation, more effective processes, such as AOPs-based scalable treatment technologies that can be retrofitted in the existing WWTPs, must be explored.

Overall, the field of LCM is still in its early stages and requires significant advancement through focused research, especially at WTPs, which can have a direct consequence on human health through drinking water consumption.

## Key References

- Feng Z, Du B, Shen M, Han X, Liang X, Zeng L. Nationwide occurrence and distribution of liquid crystal monomers in municipal sewage sludge of China. Science of The Total Environment. 2023;892:164,453.
  - This study provided the first estimation of LCM fate in wastewater treatment plants, highlighting their potential environmental impact. Additionally, a ranking of LCMs based on predicted persistence was established, offering insights into their long-term behavior in aquatic systems.
- Zhan Y, Jin Q, Lin H, Tao D, Law LY, Sun J, et al. Occurrence, behavior and fate of liquid crystal monomers in municipal wastewater. Water Res. 2023;247:120,784.
  - Based on this study it was found that LCMs with higher logKow values exhibited greater solid-water partitioning (Kd). In wastewater treatment, LCMs were primarily removed through sorption (58.4%) and degradation (25.4%).
- Wu E, Chen H, Tang L, Zeng L, Ji H, Zhu M. Molecular understanding on ultraviolet photolytic degradation of cyano liquid crystal monomers. J Hazard Mater. 2024;465.
  - The study highlighted that the photolysis of cyano LCMs is influenced by direct and photosensitized oxygenation. The excited-state properties of LCMs were simulated using theoretical methods, providing insights into their reaction mechanisms at the molecular orbital level.
- Wu J, Ye W, Feng Y, Lao W, Li J, Lu H, et al. Aquatic photolysis of high-risk fluorinated liquid crystal monomers: kinetics, toxicity evaluation, and mechanisms. Water Res. 2024;255.
  - This study highlighted the impact of UV irradiation in removing fluorinated liquid crystal monomer such as 4-cyano-3-fluorophenyl 4-ethylbenzoate (CEB-F), with O₂•⁻ and ^3^CEB-F* playing key roles in its photolysis. Possible degradation pathways and products were identified. CEB-F exhibited high toxicity to *Daphnia magna*, but UV treatment significantly reduced its toxicity.

## Supplementary Information

Below is the link to the electronic supplementary material.

Supplementary Material 1 (PDF 287 KB)

## Data Availability

No datasets were generated or analysed during the current study.
