# Supplementary material for: Liquid Crystal Monomers (LCMs) of Emerging Concern: Recent Progress and Challenges in Wastewater Treatment
Source: Curr Pollut Rep. 2025 Aug 11;11(1):48. doi: 10.1007/s40726-025-00377-3 (PMC12339604; doi:10.1007/s40726-025-00377-3)
Supplement: Supplementary file 1 — Supplementary Material 1 (PDF 287 KB) [file 40726_2025_377_MOESM1_ESM.pdf]

Liquid Crystal Monomers (LCMs) of Emerging Concern: Recent Progress and Concerns during Wastewater Treatment

Sanjeeb Mohapatra[1,*], Mui-Choo Jong[2] , Suparna Mukherji[3], Jules. B. van Lier[1], and Henri Spanjers[1]

[1]Department of Water Management, Delft University of Technology, 2628 CN Delft, The Netherlands

[2]Institute of Environment and Ecology, Tsinghua Shenzhen International Graduate School, Tsinghua University, Shenzhen 518005, China

[3]Environmental Science and Engineering Department, Indian Institute of Technology Bombay, Mumbai, India

[*]Corresponding author: s.mohapatra@tudelft.nl

Table S1: The toxicological information of Liquid crystal monomers (LCMs) obtained from *in vivo*, *in vitro*, and *in silico* studies. (Reused with permission from Wang et al., (2024))

**Test type**	**Method**	**Target LCMs**	**Species**	**Exposure time**	**Effects**	**Effect concentration**	**Reference**
				***In silico* study**			
Chronic toxicity	ECOSAR Program	330 LCMs from 10 LCM manufacturers	Fish	-	Chronically toxic to fish	Chronic toxicity values (ChV)<10 mg/L	Li et al. 2018
Acute & developmental toxicity	Toxicity Estimation Software Tool (T.E.S.T.)	Four fluorinated LCMs: TPrCB, DPrCB, BBDB, ECTB & photocatalytic degradation products	Rat	-	High acute & developmental toxicity. Some LCM intermediates showed higher toxicity, while some final products showed significantly reduced toxicity.	Oral rat LD_{50}: 41.86–562.43 mg/kg. Developmental $EC_{50:}$ 0.70–1.08 mg/kg	He et al. 2022
Acute toxicity	T.E.S.T.	12 fluorinated LCMs: EDPB, EDPBB, EDPrB, BCEDB, TrPrB, DEB, TECB, TPrCB, DECB, DPrCB, DPeCB, EBDMB & degradation products	Fathead minnow & daphnia	-	Fluorinated LCMs were classified as “very toxic” (LC_{50} < 1 mg/L). Some degradation products can be classified as “toxic” (1 mg/L< LC_{50} < 10 mg/L)	Fathead minnow LC_{50}: 0.026–7.1 mg/L. Daphnia magna LC_{50}: 0.15–0.89 mg/L	He et al. 2023
Developmental toxicity & mutagenicity	T.E.S.T.	12 fluorinated LCM & degradation products	-	-	Highly Developmental toxicity & mutagenicity, lower toxicity of degradation products	Developmental toxicity: 0.41–0.85 mg/L. Mutagenicity: 0.06–0.53 mg//L.	He et al. 2023
Acute toxicity	ECOSAR program	CEB-2F & transformation products (TPs)	Green algae, daphnia, & fish	-	Parent LCMs were classified as toxic (1 mg/L< LC_{50}/EC_{50} <10 mg/L) to aquatic organisms.	LC_{50}/EC_{50}: 1.29−4.18 mg/L.	Li et al. 2021

Chronic toxicity	ECOSAR program	CEB-2F & TPs	Green algae, daphnia, & fish	-	CEB-2F exhibited chronic toxicity to aquatic organisms. Some TPs exhibited higher acute & chronic toxicities.	ChV<1 mg/L for fish & green algae, =1.60 mg/L for daphnia	Li et al. 2021
Acute toxicity	ECOSAR & T.E.S.T software	EDPrB & hepatic metabolites	fish, daphnia, & green algae	-	EDPrB is very toxic to fish, daphnia, & green algae.	$LC_{50}/EC_5 <$ 1mg/L	Wang et al. 2023
Chronic toxicity	ECOSAR & T.E.S.T software	EDPrB & metabolites	fish, daphnia, & green algae, rat	-	EDPrB exhibit no developmental toxicity or mutagenicity, while its metabolites show developmental & mutagenic toxicity in rats.	ChV: < 1mg/L	Wang et al. 2023
Acute toxicity	ECOSAR program	m-TEB & TPs	Green algae, daphnia, & fish	-	m-TEB was very acutely toxic to three aquatic organisms	$LC_{50}/EC_{50} < 1$ mg/L	Huang et al. 2022
Chronic toxicity	ECOSAR program	m-TEB & TPs	Green algae, daphnia, & fish	-	m-TEB was very chronically toxic to fish & daphnia, & chronically toxic to green algae. Some TPs exhibited higher acute/chronic toxicity	Fish & daphnia: ChV<0.1 mg/L. Green algae: 0.1< ChV <1 mg/L	Huang et al. 2022
Acute toxicity	ECOSAR program	1173 LCMs	Green algae, daphnia, mysid, & fish (saltwater)	48 h for daphnia & 96 h for fish, mysid, algae	Acutely toxic to fish (salt water), mysids, daphnia, & green algae	Green Algae 96-hr EC_{50} =2.16 mg/L. Daphnid 48-hr LC_{50} <1 mg/L. Fish 96-hr LC_{50}: 6.10E-05–9.39E-01 mg/L. Mysid 96-hr LC_{50}: 1.14E-13–7.33 mg/L.	Su et al. 2022
Acute & Chronic toxicity	ECOSAR: acute aquatic toxicity, TEST: acute aquatic	1431 LCMs	-	-	2 % are acutely & chronically toxic to aquatic organisms. 20% are orally toxic, 33 % may cause	-	Feng et al. 2023

	toxicity, acute oral toxicity, & developmental toxicity, VEGA: developmental toxicity, skin sensitization, & mutagenicity				eye damage or skin irritation after dermal contact, & 21 % may cause respiratory irritation after inhalation. Some have developmental toxicity & organotoxicity.		
Acute & chronic aquatic toxicity	TOPKAT module, Admetsar2 website & Vega software	1173 LCMs	Algae, crustacea, & fish	-	All 1173 LCMs belonged to acute toxicity level 1, & 1157 LCMs (98.63%) belonged to chronic toxicity level 1 based on the GHS criteria	-	He et al. 2024
Carcinogenic, antagonistic, & endocrine effects	TOPKAT module, Admetsar2 website & Vega software	1173 LCMs	-	-	1151 LCMs have estrogenic effects, 1083 LCMs have androgenic effects, 101 LCMs are teratogenic, & 77 LCMs are carcinogenic, 947 LCMs can bind to PPAR-γ receptors, 1103 LCMs can bind to thyroid hormone receptors, & 430 LCMs can bind to glucocorticoid receptors	-	He et al. 2024
				***In vivo* study**			
Acute toxicity	Oral toxicity test	LCs with nitrile group, fluorinated or chlorinated groups	-	-	No acute oral toxicity, mild effect for skin irritation	LD_{50}>1000 mg/kg	Takatsu et al. 2001
Acute toxicity	Inhalation (mist) test	LCs with nitrile group, fluorinated or chlorinated groups	-	-	Greater than guideline	LC_{50}>1.0 mg/L	Takatsu et al. 2001
Acute test	Daphnia immobilization test	Ten LCs (e.g., tFBEt3cH, 5bcHdFP)	*Daphnia magna*	48 hours	None of the LCs caused immobilization to daphnia	EC_{50}>100 mg/L	Simon-Hettich et al. 2001
Acute test	Algal growth inhibition test	Ten LCs (e.g., tFBEt3cH, 5bcHdFP)	Algae *Desmodesm*	72 hours	None of the LCs caused algal growth inhibition	EC_{50}>100 mg/L	Simon-Hettich et al. 2001

			us subspicatus				
Chronic oral test	Antioxidant enzyme assays	LCs collected from 4 LCDs	Freshwater catfish	40 days	Significant inductions of CAT, SOD, Se-GPx, & GST activities	20 µg LC/g fish·day	An et al. 2008
Acute test	Acute mortality test	Four LCMs: 2O3cHdFP, tFPO-CF2-dF3B, TPrCB, BDPrB	4-6th brood offspring (<24 h old) of *Daphnia magna*	48 hours	High acute toxicity & cause morphological damage to *Daphnia magna.*	24h-LC_{50}: 0.38−13.19 mg/L<br>48h-LC_{50}: 0.04−4.32 mg/L	He et al. 2023
Chronic exposure	Reproduction inhibition test	Four LCMs: 2O3cHdFP, tFPO-CF2-dF3B, TPrCB, BDPrB	*Daphnia magna*	21 days	Disrupted thorax development, altered biomarkers (e.g., hydroxyecdysone, chitobiase, acetylcholinesterase (AChE), & antioxidant enzymes), reduced brood size, adult body length, & offspring count, indicating impaired neurotransmission, oxidative stress, endocrine disruption, molting disturbances, growth & reproduction inhibition caused by LCMs.	-	He et al. 2023
Chronic exposure	Morphological and histological observation; transcriptome sequencing and quantitative real-time PCR; T-screen assay	Six common FLCMs: 2O3cHdFP, tFPO-CF2-dF3B, 2O3cHdFB, 2OdFP3bcH, TPrCB and BDPrB	Zebrafish	144 h	Deformities; inhibited phototactic behavior; changed HPT-related hormone, enzyme, protein, and opsin content; altered genes associated with thyroid hormone-related pathways, including thyroid hormone synthesis and signaling pathways, and vision-related functions like visual perception, photoreceptor cell differentiation	2O3cHdFP: 300, 30, 3 2O3cHdFP; tFPO-CF2-dF3B: 100, 10, 1 ng/L; 2O3cHdFB: 45, 4.5, 0.45 ng/L; 2OdFP3bcH: 60, 6, 0.6 ng/L; TPrCB: 50, 5, 0.5 ng/L; BDPrB: 10, 1, 0.1 ng/L	He et al. 2024

Acute toxicity test	Viability bioassay (bacterial growth) Fluorescence assay (bacterial membrane permeability)	Lyotropic chromonic LCs (neutral grey, red 14, blue 27, cromolyn), surfactant-based lyotropic LCs (CPCl, CsPFO) & thermotropic LCs (5CB, E7)	Three types of bacteria: *Staphylococcus aureus*, *Bacillus atrophaeus* & *Escherichia coli*	15 min	Lyotropic chromonic LCs showed no effect on the growth & survival of three bacteria. Surfactant-based lyotropic LCs (CPCl & CsPFO) significantly inhibited the growth of *E. coli* & *S. aureus*, thermotropic LCs (5CB & E7) significantly inhibited germination & growth of *B. atrophaeus*. *E. coli* treated with surfactant-based lyotropic LCs showed 85–90% dead.	-	Woolverton et al. 2005
Acute & chronic	Microbial growth, metabolome, enzymatic activity, & mRNA expression	8 commercial LCMs	Two representative human commensal bacteria: *Escherichia coli* (*E. coli*) & *Staphylococcus epidermidis* (*S. epidermidis*)		Growth inhibition, dysregulated fatty acid metabolism, oxidative stress	Low: 10 ng/mL; high: 1000 ng/mL	Huang et al. 2024
**In vitro study**							
Mutagenic toxicity test	Ame’s test	LCs with nitrile group, fluorinated or chlorinated groups	-	-	Negative, no mutagenicity	-	Takatsu et al. 2001
Acute test	Dual fluorescent staining (viability & cytotoxicity) assays	LCs containing phenyl, cyclohexyl, ether, ester, & cyano groups & LCs containing fluorophenyl groups	Mammalian cell lines—3T3 fibroblast & SV-40 transformed human	4 hours	Most LCs with phenyl, cyclohexyl, ether, ester, & cyano groups can cause cell death & exhibited cytotoxicity, LCs with fluorophenyl groups showed minimal or no lethal toxicity	-	Luk et al. 2004

			corneal epithelial cells (HCEC)				
Acute lethal test	Cell viability assay	Six LCMs: 2OdF3B, tFPO-CF2-tF3T, 2teFT, 5OCB, 6OCB, 8OCB	Human kidney 2 (HK2) cell	24 hours	Most LCMs posed no lethal threat, but 100 μM 2OdF3B caused a 22.9% reduction in the HK2 cell survival rate.	Concentration gradient: 0, 20, 40, 60, 80 & 100 μM	Zhao et al. 2023
Sublethal test	Metabolomics analysis	Three LCMs: 2OdF3B, tFPO-CF2-tF3T, 8OCB	HK2 cell	24 hours	56 significantly dysregulated metabolites, including nucleoside phosphates, amino acids, fatty acids, organooxygen/organonitrogen compounds, & glycerophospholipids.	20 μM 2OdF3B, 100 μM tFPO-CF2-tF3T & 8OCB	Zhao et al. 2023
Sublethal test	Transcriptomics analysis	8OCB	HK2 cell	24 hours	1817 significant differentially expressed genes, the top enriched functions in biological processes are related to cell apoptosis.	100 μM	Zhao et al. 2023
Toxicogenomic test	Avian ToxChip PCR array or targeted real-time PCR	6 LCM mixtures from 6 frequently used smartphone LCD panels	Chicken embryonic hepatocytes (CEHs)	24 hours	Five genes, *CYP1A4, FGF19, LBFABP, PDK4,* & *THRSP*, were significantly dysregulated	2 units per mL	Su et al. 2019
Acute cell viability	Cell Counting Kit-8 method	14 LCMs (2O3cHdFB, tFPO-CF2-dF3B, 2O3cHdFP, 2O2cHdFB, TePT, 2F3T, tFMePO-CF2-dF3B, DMPMB, MeO3bcH, DPrB, 3cH2B, 2OdF3B, tFPO-CF2-tF3T, MeP3bcH) & LCM mixture	Human lung bronchial epithelial BEAS-2B cells	24 hours	0.34 μg/mL LCM & mixed LCMs did not significantly reduce BEAS-2B cell viability, 3.4 & 34 μg/mL LCMs significantly decreased cell viability with a dose-dependent cytotoxicity	Concentration ranges of 0.34−34 μg/mL	Jin et al. 2023

Sublethal effect	Human ROS ELISA	14 LCMs & LCM mixture	Human lung bronchial epithelial BEAS-2B cells	24 hours	Certain LCMs induced ROS generation at relatively low dosage (e.g., 1.02 μg/mL), indicating oxidative stress caused by LCMs	Concentration ranges of 1.02−10.2 μg/mL	Jin et al. 2023
			***In silico* & *in vitro* integrated study**				
Sublethal test	Inverse virtual screen & reporter gene assay	38 LCMs for *in silico* assays, six LCMs (2OdF3B, tFPO-CF2-tF3T, 2teFT, 5OCB, 6OCB, 8OCB) for reporter cell assays	GeneBLAzer cell lines, PPARγ-UAS-bla HEK 293H cells, Erα-UAS-bla-GripTite & GR-UAS-bla HEK 293T	18 hours	LCMs can bind to PPARγ with antagonist effects. Dysregulated fatty acid oxidation might be mediated through multiple pathways (IL-17, TNF, NF-kB, AMPK) & ligand-dependent PPARγ antagonism.	The effective concentration causing a suppression ratio of 20% ($ECSR_{20}$) value: 10.4−88.3 μM	Zhao et al. 2023
Mutagenicity	*in vitro* mammalian chromosome aberration test, bacterial reverse mutation assay, T.E.S.T. software, VEGA platform	16 LCMs detected in obsolete smartphone screen	-	-	All 16 LCMs were shown to be non-mutagenic based on the *in vitro* test & *in silico* prediction.	-	Feng et al. 2022
Carcinogenicity	Four models (CAESAR,35 ISS, IRFMN/Antares, & IRFMN/ISSCANC GX models) in VEGA platform	16 LCMs detected in obsolete smartphone screen	-	-	Experimental data showed no carcinogenicity. 12 LCMs were predicted to be carcinogenic by VEGA.	-	Feng et al. 2022

Table S2: Physical and chemical properties of selected LCMs shown in Figure 1

Abbreviation (Abbr.)	CASRN[a]	Name	Mole. Wt. (g/mol)	Structural formula	Boiling Point (℃)	logK_{ow}[b]	logKoa[c]	P_L[d]
MOPrCHB	81936-32-5	1-Methoxy-4-(trans-4-propylcyclohexyl)benzene	232.36	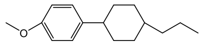	361.49	6.29	7.502	1.26×10^{-1}
2OdF3B	157248-24-3	1-ethoxy-2,3-difluoro-4-(4-propylphenyl)benzene	276.3	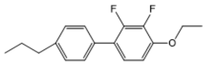	409.65	6.26	8.71	1.81×10^{-2}
EPhEMOB	63221-88-5	1-(2-(4-ethylphenyl)ethynyl)-4-methoxybenzene	236.31	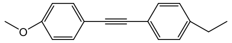	410.15	5.14	8.87	1.85×10^{-2}
3OCB	52709-86-1	4'-Propoxy-4-biphenylcarbonitrile	237.3	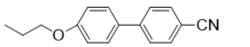	480.35	4.37	8.825	3.69×10^{-3}
DTMDEB	303186-19-8	4-[Difluoro(3,4,5-trifluorophenoxy)methyl]-3,5-difluoro-4'-ethyl-biphenyl	414.32	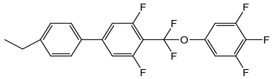	535.91	8.61	10.09	1.63×10^{-3}
2teFT	326894-55-7	4''-Ethyl-2',3,4,5-tetrafluoro-1,1':4',1''-terphenyl	330.3	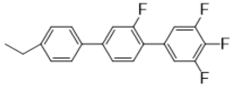	491.05	7.36	8.97	1.43×10^{-3}
TrPrB	132123-39-8	3,4,5-Trifluoro-4'-(trans-4-propylcyclohexyl)biphenyl	332.41	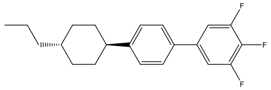	492.9	8.57	8.636	2.87×10^{-3}
DPrB	85312-59-0	3,4-Difluoro-4'-(trans-4-propylcyclohexyl)biphenyl	314.42	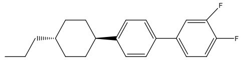	488.65	8.37	8.899	2.47×10^{-3}
TePT	205806-87-7	2',3,4,5-Tetrafluoro-4''-propyl-1,1':4',1''-terphenyl	344.35	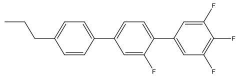	513.93	7.85	9.337	7.45×10^{-4}
tFMePO-CF$_2$-Df3b	1690317-23-7	4-[difluoro(2-methyl-3,4,5-trifluorophenoxy)methyl]-3,5-difluoro-4'-propylbiphenyl	442.4	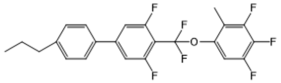	586.65	9.65	9	1.82×10^{-5}
3cH2B	84540-37-4	1-ethyl-4-(4-(4-propylcyclohexyl)phenyl)benzene	306.5	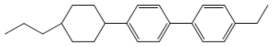	530.89	9.01	10.08	5.00×10^{-4}
2F3T	95759-44-7	4''-ethyl-2'-fluoro-4-propyl-1,1':4',1''-terphenyl	318.4	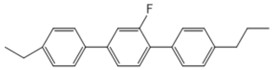	551.92	8.29	10.44	1.28×10^{-4}

2F4T	825633-75-8	4-butyl-4’’-ethyl-2’-fluoro-1,1’:4’,1’’-terphenyl	332.5	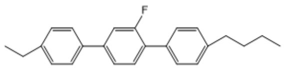	574.8	8.78	11.02	6.59×10^{-5}
3bcHdFB	119990-81-7	3,4-difluoro-4’-[4’-propyl-1,1’-bi(cyclohexyl)-4-yl]biphenyl	396.6	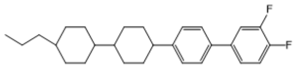	640.81	11.06	11.64	4.38×10^{-5}

a. CASRN: Chemical abstracts service registry number.

b. logK_{ow}: the octanol-water partition coefficient.

c. logK_{oa}: the octanol–air partitioning coefficient.

References

An, R., Y. Li, X. Niu and H. Yu (2008). "Responses of antioxidant enzymes in catfish exposed to liquid crystals from E-waste." Int J Environ Res Public Health **5**(2): 99-103.

Feng, J. J., X. F. Sun and E. Y. Zeng (2022). "Emissions of Liquid Crystal Monomers from Obsolete Smartphone Screens in Indoor Settings: Characteristics and Human Exposure Risk." Environ Sci Technol **56**(12): 8053-8060.

Feng, J. J., X. F. Sun and E. Y. Zeng (2023). "Predicted health and environmental hazards of liquid crystal materials via quantitative structure-property relationship modeling." J Hazard Mater **446**: 130592.

He, S., J. He, F. Wu, Y. Zhao, X. Jin and C. J. Martyniuk (2023). "In vivo and in silico toxicity assessment of four common liquid crystal monomers to Daphnia magna: Novel endocrine disrupting chemicals in crustaceans?" Sci Total Environ **912**: 168757.

He, S., M. Shen, E. Wu, R. Yin, M. Zhu and L. Zeng (2022). "Molecular structure on the detoxification of fluorinated liquid crystal monomers with reactive oxidation species in the photocatalytic process." Environ Sci Ecotechnol **9**: 100141.

He, S., E. Wu, M. Shen, H. Ji, L. Zeng and M. Zhu (2023). "Role of Substituents in the Removal of Emerging Fluorinated Liquid Crystal Monomer Pollutants under the UV/Peroxydisulfate Treatment." ACS ES&T Engineering **3**(5): 651-660.

He, W., Y. Cui, H. Yang, J. Gao, Y. Zhao, N. Hao, Y. Li and M. Zhang (2024). "Aquatic toxicity, ecological effects, human exposure pathways and health risk assessment of liquid crystal monomers." J Hazard Mater **461**: 132681.

Huang, Y., Q. Ruan, S. Fang, Y. Duan, J. Zheng, Z. Xiang, Y. Shen, S. Liu and G. Ouyang (2024). "Toxicity Assessment of Environmental Liquid Crystal Monomers: A Bacteriological Investigation on Escherichia coli and Staphylococcus epidermidis." Environmental Science & Technology **58**(7): 3141-3150.

Huang, Y., X. Zhang, C. Li, Y. Zhao, Y. N. Zhang and J. Qu (2022). "Atmospheric persistence and toxicity evolution for fluorinated biphenylethyne liquid crystal monomers unveiled by in silico methods." J Hazard Mater **424**(Pt B): 127519.

Jin, Q., Y. Fan, Y. Lu, Y. Zhan, J. Sun, D. Tao and Y. He (2023). "Liquid crystal monomers in ventilation and air conditioning dust: Indoor characteristics, sources analysis and toxicity assessment." Environ Int **180**: 108212.

Li, C., Y. Huang, X. Zhang, Y. Zhao and Y. Huo (2021). "Atmospheric Fate and Risk Investigation of Typical Liquid Crystal Monomers." ACS Sustainable Chemistry & Engineering **9**(9): 3600-3607.

Li, J., G. Su, R. J. Letcher, W. Xu, M. Yang and Y. Zhang (2018). "Liquid Crystal Monomers (LCMs): A New Generation of Persistent Bioaccumulative and Toxic (PBT) Compounds?" Environ Sci Technol **52**(9): 5005-5006.

Luk, Y.-Y., S. F. Campbell, N. L. Abbott and C. J. Murphy (2004). "Non-toxic thermotropic liquid crystals for use with mammalian cells." Liquid Crystals **31**(5): 611-621.

Simon-Hettich, B., T. Broschard, W. Becker, H. Takeuchi, H. Saito, H. Ohnishi, H. Takatsu, S. Naemura and K. Kobayashi (2001). "Ecotoxicological properties of liquid-crystal compounds." Journal of The Society for Information Display - J SOC INF DISP **9**.

Su, H., K. Ren, R. Li, J. Li, Z. Gao, G. Hu, P. Fu and G. Su (2022). "Suspect Screening of Liquid Crystal Monomers (LCMs) in Sediment Using an Established Database Covering 1173 LCMs." Environ Sci Technol **56**(12): 8061-8070.

Su, H., S. Shi, M. Zhu, D. Crump, R. J. Letcher, J. P. Giesy and G. Su (2019). "Persistent, bioaccumulative, and toxic properties of liquid crystal monomers and their detection in indoor residential dust." Proc Natl Acad Sci U S A **116**(52): 26450-26458.

Takatsu, H., H. Ohnishi, K. Kobayashi, W. Becker, M. Seki, M. Tazume, T. Nakajima, H. Saito, B. Simon-Hettich and S. Naemura (2001). "Investigation Activity and Data on Safety of Liquid Crystal Materials." Molecular Crystals and Liquid Crystals Science and Technology. Section A. Molecular Crystals and Liquid Crystals **364**(1): 171-186.

Wang, X., R. Yang, J. Zhang, X. Chen, Y. Feng, Y. Niu and B. Shao (2023). "Metabolic profiling of the fluorinated liquid-crystal monomer 1-ethoxy-2,3-difluoro-4-(trans-4-propylcyclohexyl)benzene." Sci Total Environ **860**: 160448.

Wang, Y., Jin, Q., Lin, H., Xu, X., Leung, K. M., Kannan, K., & He, Y. (2024). "A review of liquid crystal monomers (LCMs) as emerging contaminants: Environmental occurrences, emissions, exposure routes and toxicity." J Hazard Mater, 480, 135894.

Woolverton, C. J., E. Gustely, L. Li and O. D. Lavrentovich (2005). "Liquid crystal effects on bacterial viability." Liquid Crystals **32**(4): 417-423.

Zhao, H., C. Li, M. Y. Naik, J. Wu, A. Cardilla, M. Liu, F. Zhao, S. A. Snyder, Y. Xia, G. Su and M. Fang (2023). "Liquid Crystal Monomer: A Potential PPARγ Antagonist." Environ Sci Technol **57**(9): 3758-3771.
